# Perforated Gangrenous Appendicitis Despite a Normal Leukocyte Count and Nondiagnostic Ultrasonography in a 5‐Year‐Old Child A Case Report

**DOI:** 10.1002/ccr3.73849

**Published:** 2026-11-02

**Authors:** Iswor Karki, Sagar Kumar Jha, Shankar Poudel, Sabitra Thapa, Latika Giri, K. C. Ajay

**Affiliations:** ^1^ Institute of Medicine Tribhuvan University Teaching Hospital, Tribhuvan University Kathmandu Nepal; ^2^ Department of Surgery Kathmandu University School of Medical Sciences Dhulikhel Nepal; ^3^ Department of Surgery B&C Medical College Teaching Hospital and Research Center Birtamode Nepal

**Keywords:** case report, nondiagnostic ultrasonography, normal leukocyte count, patent ductus arteriosus, pediatric appendicitis, perforated appendicitis

## Abstract

A 5‐year‐old girl with prior patent ductus arteriosus device closure presented with abdominal pain, vomiting, and fever. Despite a normal leukocyte count and nonvisualization of the appendix on ultrasonography, computed tomography identified perforation. Emergency appendectomy confirmed gangrenous appendicitis. This case underscores the importance of serial examination and timely diagnostic imaging.

## Key Clinical Message

A normal leukocyte count and a nonvisualized appendix on ultrasonography do not exclude perforated appendicitis in young children. Persistent clinical concern and evolving abdominal signs warrant reassessment and appropriate further imaging. Previous patent ductus arteriosus (PDA) device closure should prompt proportionate cardiac assessment without distracting from the acute surgical diagnosis.

## 1 Introduction

Appendicitis in young children can be difficult to recognize before complications develop. Symptoms may overlap with common childhood illnesses, and examination findings can evolve during observation [1, 2]. Studies of young children and of complicated pediatric appendicitis highlight the importance of interpreting age, symptom history, examination, and investigations together. A normal leukocyte count reduces concern in some clinical settings but cannot independently exclude appendicitis [3]. Likewise, failure to visualize the appendix on ultrasonography does not establish a normal appendix; the clinical course and accompanying imaging findings remain relevant [4].

A history of repaired congenital heart disease adds a separate assessment task before emergency surgery. Perioperative risk depends on the residual physiology, ventricular function, pulmonary vascular disease, and the planned procedure, rather than the diagnostic label alone [5]. Transcatheter patent ductus arteriosus (PDA) closure is an established intervention with device‐specific technical and follow‐up considerations [6].

We report perforated gangrenous appendicitis in a 5‐year‐old child with a normal leukocyte count and a nonvisualized appendix on initial ultrasonography. The central teaching point is the diagnostic importance of continued clinical assessment and further imaging. The prior PDA closure also provides context for discussing proportionate perioperative assessment. The report is structured with reference to the Surgical Case Report (SCARE) 2023 guideline [7].

## 2 Case Presentation

### 2.1 Presentation and Cardiac History

A 5‐year‐old girl weighing 18 kg presented to the emergency department of Tribhuvan University Teaching Hospital, Kathmandu, Nepal, with a 1 day history of abdominal pain. The pain began in the periumbilical region and migrated to the right lower abdomen. She had six to seven episodes of vomiting, anorexia, and fever, with a maximum recorded temperature of 100.5°F (approximately 38.1°C). There was no history of diarrhea, dysuria, or jaundice.

Eleven months earlier, she had undergone transcatheter device closure of a PDA associated with left atrial and left ventricular dilatation at the same institution. The device model and manufacturer, date of the latest echocardiogram, and quantitative follow‐up cardiac measurements were not available for this report. Her aspirin or other antiplatelet medication status could not be established from the available information.

At presentation, she was conscious and hemodynamically stable, with no pallor, icterus, cyanosis, or clinical dehydration. Heart sounds were normal, and no murmur was heard. Abdominal examination demonstrated tenderness in the right iliac fossa, right flank, and right hypochondrium. Progressive muscle guarding developed on serial assessment, increasing concern for peritoneal inflammation.

### 2.2 Investigations and Diagnostic Assessment

The total leukocyte count was 8200 cells/μL, hemoglobin was 12.8 g/dL, and platelet count was 165,000/μL. Renal function, electrolytes, and urine examination were reported as unremarkable. C‐reactive protein and differential leukocyte results were not available for this report. The initial differential diagnosis included appendicitis, mesenteric adenitis, and viral gastroenteritis.

Abdominal and pelvic ultrasonography did not visualize the appendix. It identified several enlarged periumbilical lymph nodes, the largest measuring 8 × 17.5 mm, and free fluid between bowel loops. These findings were nonspecific but did not resolve the clinical concern. Subsequent contrast‐enhanced CT demonstrated a localized right lower quadrant hypodense collection adjacent to the partially filled urinary bladder (Figure 1), an appendicolith near the appendiceal base, a mid‐appendiceal diameter of 13.1 mm, and intraluminal air foci (Figure 2). Coronal images were reported to show tip perforation, cecal wall thickening, and enhancing mesenteric lymph nodes along the superior mesenteric vessels (Figure 3).

**FIGURE 1:**
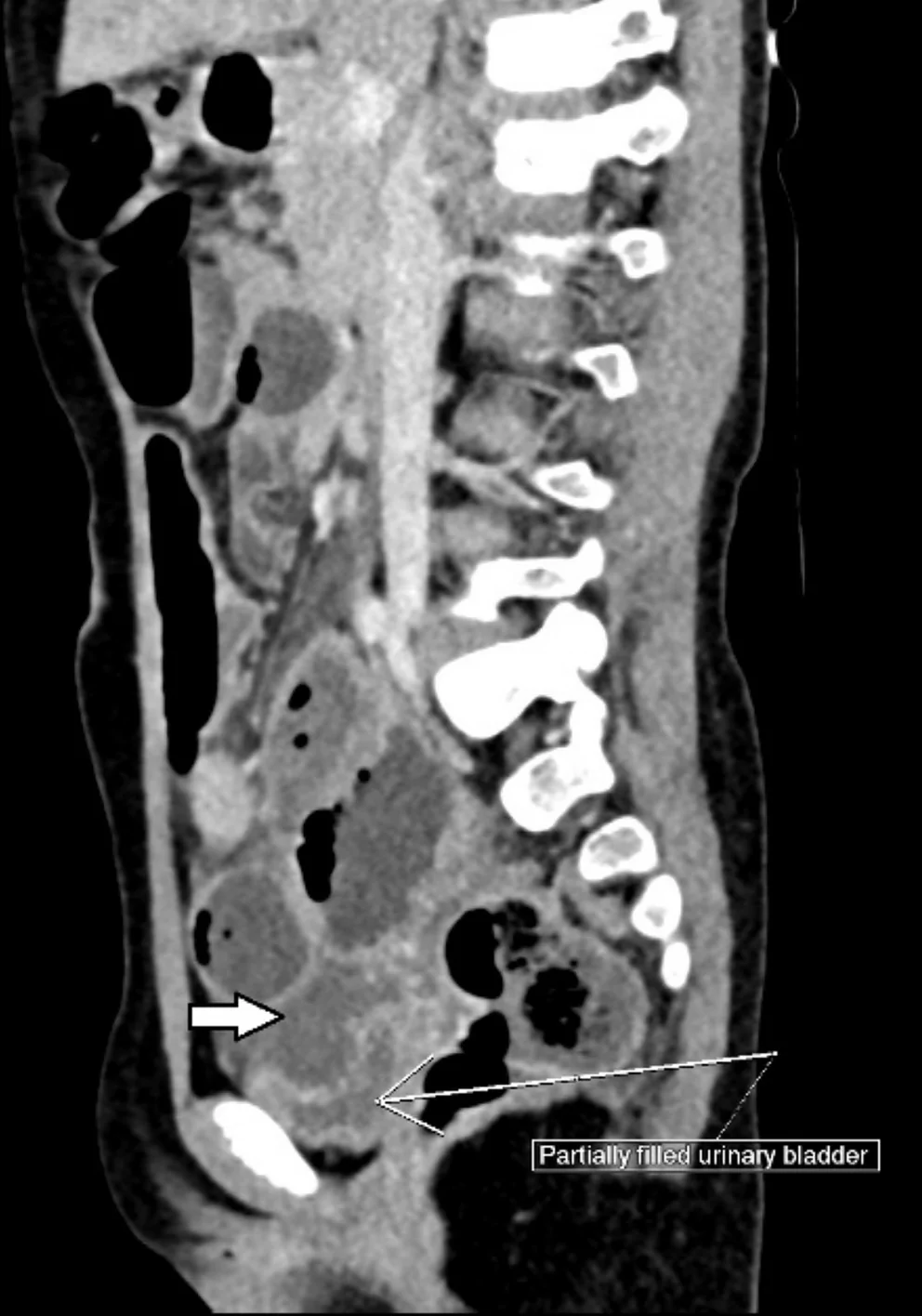
Contrast‐enhanced CT demonstrating a localized right lower quadrant hypodense collection adjacent to the partially filled urinary bladder.

**FIGURE 2:**
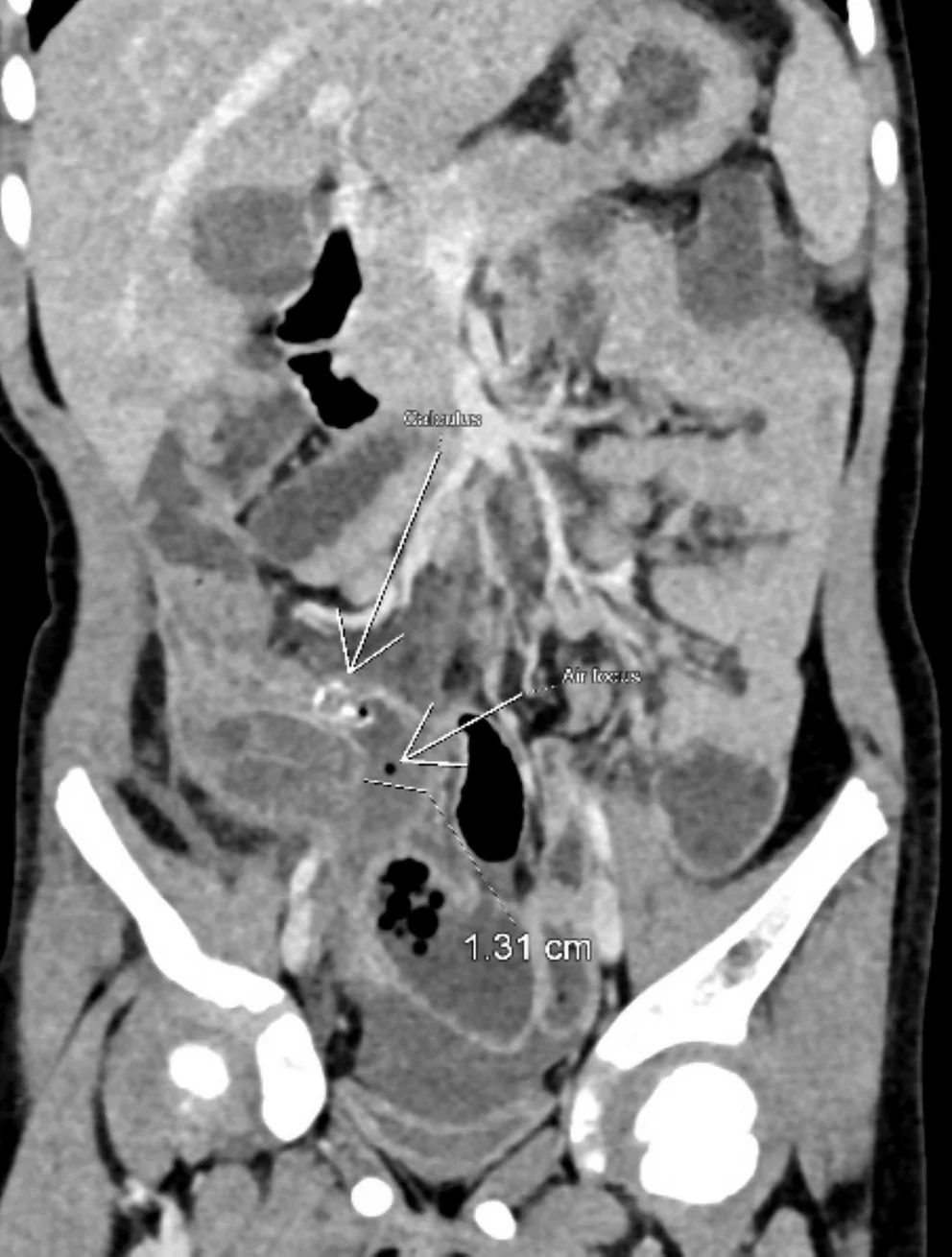
CT findings of an appendicolith near the appendiceal base, appendiceal dilatation to 13.1 mm at the mid portion, and intraluminal air foci.

**FIGURE 3:**
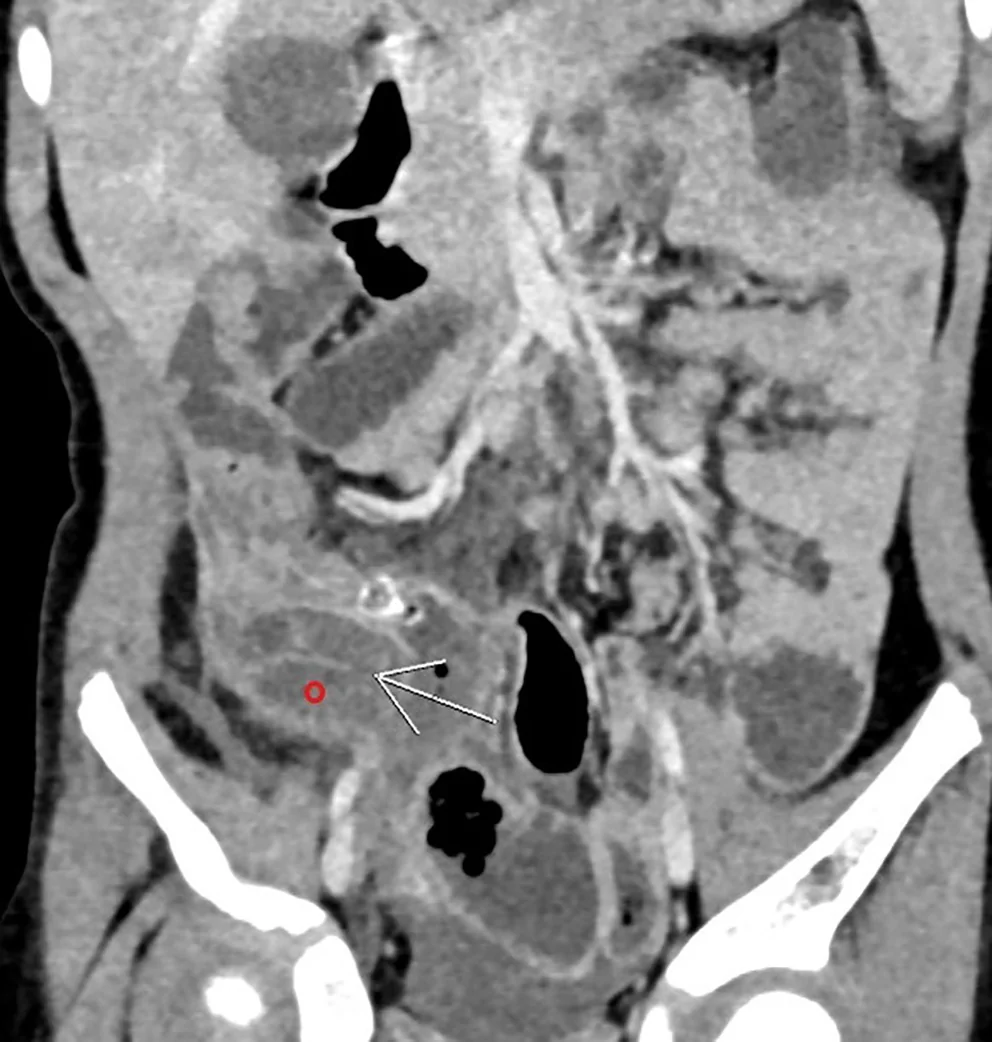
Coronal CT demonstrating reported appendiceal tip perforation, cecal wall thickening, and enhancing mesenteric lymph nodes.

Ultrasonography preceded CT, and appendectomy was performed on the day of admission. The available account also documents progressive guarding during serial examinations; however, exact assessment times and the timing of guarding relative to the CT decision were not recorded in the material available for this report.

### 2.3 Perioperative Assessment and Treatment

The child was kept nil by mouth and received intravenous dextrose normal saline with potassium chloride at a recorded rate of 58 mL/h. The recorded medications were intravenous amoxicillin‐clavulanate 675 mg, metronidazole 350 mg, ketorolac 15 mg, and paracetamol 250 mg; dosing intervals were not available. Cefotaxime 500 mg was recorded as perioperative surgical prophylaxis.

Pediatric cardiology was consulted before surgery. The assessment was reported to identify no residual cardiac defect and normal oxygen saturation on room air, and the child was considered suitable for emergency general anesthesia. The underlying echocardiographic report, residual‐shunt measurements, left heart remodeling, ventricular function, estimated pulmonary pressure, and ECG findings were not available for independent description. Normal oxygen saturation and the absence of a murmur were therefore not used as proof of complete anatomical closure.

Emergency open appendectomy through a Lanz incision was performed under general anesthesia with endotracheal intubation. Anesthetic agents, intraoperative monitoring modalities, ventilation settings, and detailed hemodynamic records were unavailable. Approximately 20 mL of periappendiceal pus and fibrinous exudate were encountered. The appendix was gangrenous, with perforation at the tip and a healthy base. Appendectomy and saline peritoneal lavage were performed, a 20 Fr pelvic drain was placed, and the abdominal wall was closed in layers. The reason for selecting an open approach was not documented in the available account.

The appendix was submitted for histopathology and the pus for culture and sensitivity testing. Gross examination described a 7.0 × 2.1 × 0.1 cm specimen with a dull, gray‐brown external surface and a fecalith within a dilated lumen (Figure 4). Microscopy showed mucosal ulceration, dense neutrophilic infiltration extending into the muscularis propria, mural necrosis, and periappendiceal inflammation, consistent with acute suppurative appendicitis (Figure 5). Gangrene and tip perforation were documented operatively. The pus culture result was not available for this report.

**FIGURE 4:**
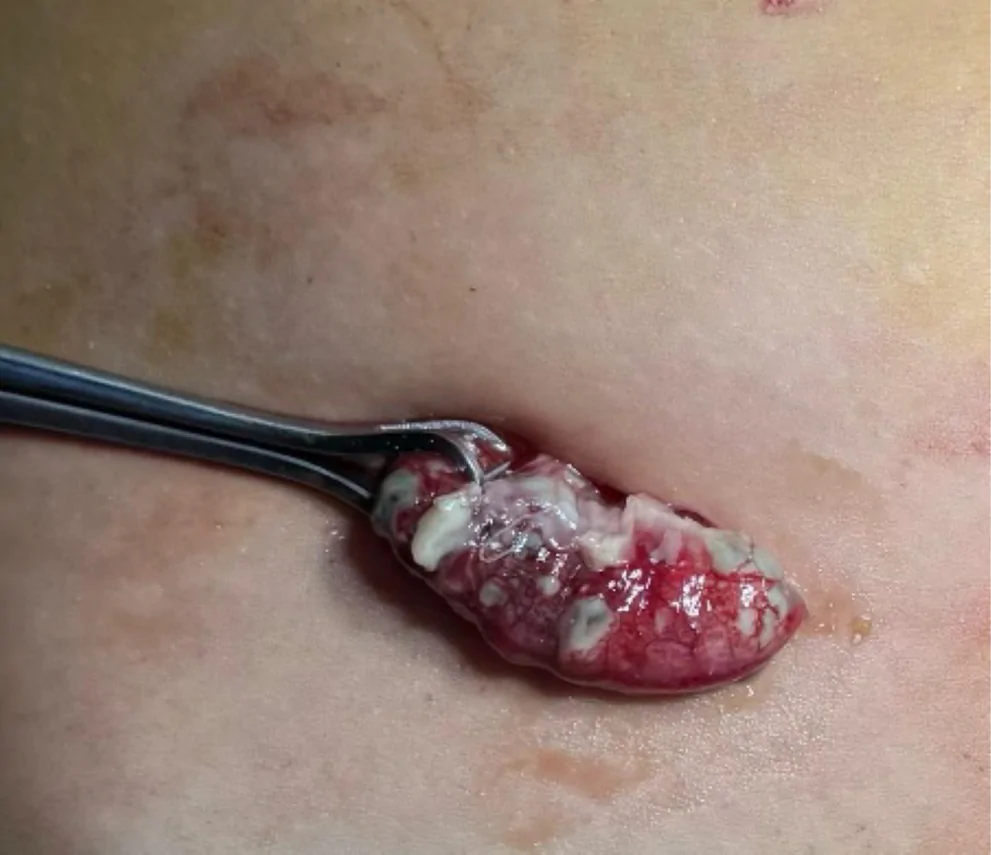
Gross appendiceal specimen with a dull, gray‐brown external surface and a fecalith within the dilated lumen.

**FIGURE 5:**
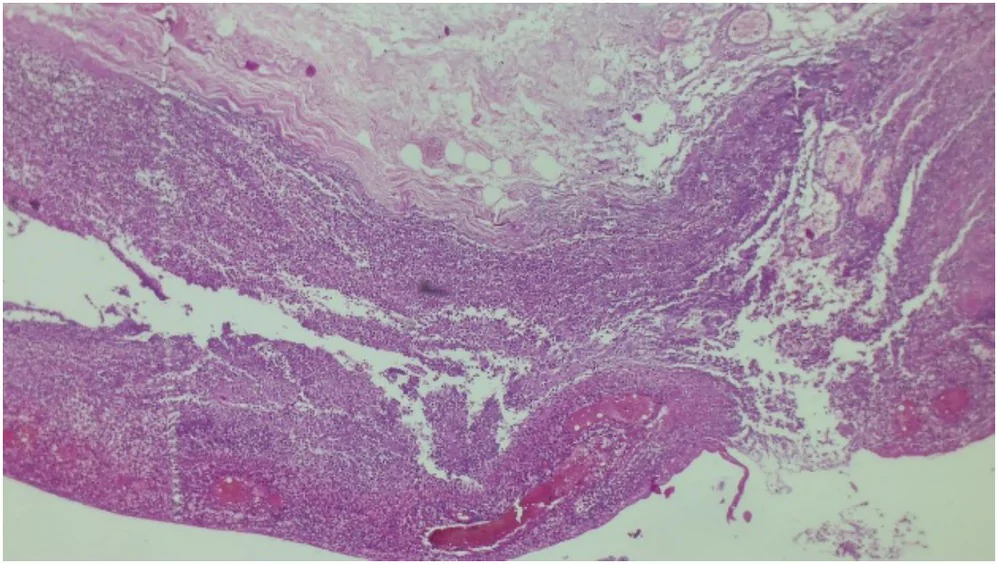
Histopathological findings of mucosal ulceration, dense neutrophilic inflammation involving the muscularis propria, mural necrosis, and periappendiceal inflammation.

### 2.4 Outcome and Follow‐Up

The reported postoperative course was uneventful. The pelvic drain was removed on postoperative day four. The child tolerated a normal diet before discharge on postoperative day seven. Oral cefixime and paracetamol were prescribed for 3 days, with paracetamol subsequently as needed. The available information did not establish the complete inpatient antibiotic course or the concentrations of the discharge syrups. Follow‐up was arranged in the pediatric surgery outpatient department with the histopathology report. Attendance and subsequent clinical findings were not documented in the information available for this report. No postoperative echocardiographic findings or cardiac images were available.

## 3 Discussion

The clinically useful feature of this case is the discordance between a normal leukocyte count and operative evidence of gangrenous perforation. The laboratory result did not match the severity of the eventual surgical findings. The history of migratory pain and the development of guarding maintained concern for appendicitis despite the absence of leukocytosis. Studies of pediatric appendicitis support combining laboratory findings with the examination rather than using a normal count as an exclusion criterion [1, 2, 3]. This case illustrates that principle; it does not estimate the frequency of perforation with a normal count.

The family reported 1 day of symptoms, but this does not establish the precise biological onset of appendiceal inflammation or the moment of perforation. Accordingly, the case should not be interpreted as proof that an initially uncomplicated appendix progressed to perforation within a measured 24 h interval.

Ultrasonography is useful in pediatric assessment, but its performance depends in part on whether the appendix is visualized and on local experience. In a multicenter cohort, sensitivity was substantially higher when the appendix was clearly identified [8]. A nonvisualized appendix should therefore be interpreted as an incomplete observation, with its significance determined by the rest of the study and the clinical assessment. The reason for nonvisualization in this patient was not documented and cannot be attributed specifically to bowel gas.

Cohen and colleagues found that the negative predictive value of nondiagnostic ultrasound increased among children without leukocytosis [4] Nah and colleagues similarly reported a low likelihood of appendicitis when a nonvisualized appendix was accompanied by no secondary inflammatory signs [9]. These findings support selective observation in suitable patients; they do not make normal WBC and nonvisualization an infallible rule‐out combination. In the present case, free fluid, persistent localized tenderness, and progressive guarding prevented an otherwise reassuring interpretation. Enlarged nodes alone could not establish mesenteric adenitis as the explanation for the whole presentation.

The American College of Radiology identifies MRI and contrast‐enhanced CT as appropriate options after an equivocal or nondiagnostic ultrasound when clinical suspicion persists [10]. A staged ultrasound‐to‐MRI pathway can also achieve good diagnostic performance while avoiding radiation [11]. Here, CT provided evidence of a periappendiceal collection and perforation after the initial ultrasound was inconclusive. The choice between repeat ultrasound, MRI, and CT depends on the clinical situation and local access. MRI availability, sedation requirements, and the exact reason CT was selected were not documented for this patient. The practical lesson is to resolve persistent diagnostic uncertainty promptly, rather than to perform CT routinely after every nonvisualized appendix.

Open appendectomy provided source control in this child, and the operative findings explained the evolving abdominal signs. This favorable outcome does not establish superiority of an open approach or prove that lavage and drainage were necessary. The 2020 WSES guideline favors laparoscopy where appropriate expertise and equipment are available; it also questions routine irrigation and prophylactic drainage, with relevant pediatric drainage evidence largely drawn from laparoscopic surgery [12].

Antibiotics in this episode addressed the abdominal infection and perioperative surgical requirements. Selection and duration should be evaluated against source control, clinical recovery, microbiology, and local protocols. The 2020 WSES guideline supports an early switch to oral therapy and a total course shorter than 7 days in children with complicated appendicitis when clinically appropriate [12]. Pediatric pathway studies have also examined outcomes and resource use with standardized management [13, 14]. Because the full inpatient antibiotic schedule and culture result are unavailable, this case cannot establish the appropriateness of the total duration or endorse the reported combination. Discharge on day seven alone does not reveal how many days of intravenous treatment were given.

The repaired PDA was a reason to review cardiovascular status, but the available account does not demonstrate a specific cardiac complication or an alteration in the operative technique. The AHA scientific statement on children with congenital heart disease undergoing noncardiac procedures provides a relevant framework for assessing residual physiology and planning care [5]. In this patient, the reported cardiology opinion was reassuring, but the lack of quantitative echocardiography and anesthesia records limits assessment of how that opinion was reached and implemented.

The manufacturer and occluder model remain unknown. Thanopoulos and colleagues described technical considerations and follow‐up with the Amplatzer duct occluder [6], but citing this publication cannot establish that an Amplatzer device was used here. Nor can an absent murmur or normal oxygen saturation substitute for documentation of device position, residual shunting, ventricular function, and pulmonary pressure. These missing data limit device‐specific interpretation.

Endocarditis prophylaxis requires consideration of both the cardiac substrate and the procedure. AHA guidance identifies completely repaired congenital defects with prosthetic material during the first 6 months after repair, and repaired defects with relevant residual abnormalities, among the higher‐risk categories for indicated dental prophylaxis [15, 16]. The AHA does not recommend antibiotics solely to prevent endocarditis for gastrointestinal procedures; treatment of an established infection is a separate issue [15]. The 2023 ESC guideline allows consideration of prophylaxis for selected high‐risk patients undergoing invasive nondental procedures, including gastrointestinal procedures, as a class IIb recommendation [17].

In this child, device closure had occurred 11 months earlier, beyond the 6‐month period, and cardiology reportedly found no residual defect. Those reported findings do not establish a separate high‐risk indication for additional endocarditis prophylaxis solely because of the prior PDA device. This interpretation remains limited by the absence of the supporting cardiac report. Antibiotics administered for perforated appendicitis should not be relabeled as device‐specific endocarditis prophylaxis.

Infection involving a PDA occluder has been reported. Yilmazer and Meşe described a child who developed fever and *Staphylococcus aureus* bacteremia 1 week after Amplatzer Duct Occluder II placement, ultimately requiring device removal [18]. This establishes the possibility of device infection but does not indicate that it occurred in the present case, 11 months after closure. Localized appendiceal pus is not evidence of bacteremia or infection of the cardiac device. The available account does not establish sepsis with organ dysfunction, bloodstream infection, or endocarditis. Without postoperative cardiac imaging and longer follow‐up, the report cannot document long‐term device status.

This single case illustrates a diagnostic principle rather than a new association between PDA closure and appendicitis. The exact sequence of repeat examinations relative to CT, inflammatory markers beyond the total leukocyte count, culture findings, complete antimicrobial course, and post‐discharge outcomes were unavailable. Device identification, detailed echocardiography, antiplatelet history, ECG findings, and anesthesia records were also unavailable. These gaps restrict conclusions about cardiac risk and perioperative decision‐making. The supported clinical message remains that normal initial laboratory findings and incomplete appendix visualization must be reconciled with the evolving examination.

## 4 Conclusion

Perforated gangrenous appendicitis can occur in a young child despite a normal leukocyte count and nonvisualization of the appendix on ultrasound. Continued assessment and appropriate further imaging helped establish the diagnosis in this case, followed by operative source control and an uneventful inpatient recovery. Previous PDA device closure was relevant to preoperative assessment, but the available evidence does not show that it created unusual management difficulties or caused a device‐related complication.

## Author Contributions

**Iswor Karki:** conceptualization, investigation, writing – original draft, writing – review and editing, resources. **Sagar Kumar Jha:** supervision, writing – original draft, writing – review and editing, project administration. **Shankar Poudel:** writing – review and editing, writing – original draft, conceptualization. **Sabitra Thapa:** investigation, visualization, project administration, writing – original draft. **Latika Giri:** writing – review and editing, writing – original draft, conceptualization. **K. C. Ajay:** resources, project administration, investigation, writing – original draft.

## Funding

The authors have nothing to report.

## Ethics Statement

The authors have nothing to report.

## Consent

Written informed consent for publication of this case report and accompanying images was obtained from the patient's legal guardian. A copy is available for review by the Editor‐in‐Chief on request.

## Conflicts of Interest

The authors declare no conflicts of interest.

## Data Availability

The data that support the findings of this study are available on request from the corresponding author. The data are not publicly available due to privacy or ethical restrictions.

## References

[1] M. L. Nance , W. T. Adamson , and H. L. Hedrick , “Appendicitis in the Young Child: A Continuing Diagnostic Challenge,” Pediatric Emergency Care 16, no. 3 (2000): 160–162, 10.1097/00006565-200006000-00005. 10888451

[2] X. B. D. Pham , V. F. Sullins , D. Y. Kim , et al., “Factors Predictive of Complicated Appendicitis in Children,” Journal of Surgical Research 206, no. 1 (2016): 62–66, 10.1016/j.jss.2016.07.023. 27916376

[3] L. T. Wang , K. A. Prentiss , J. Z. Simon , D. P. Doody , and D. P. Ryan , “The Use of White Blood Cell Count and Left Shift in the Diagnosis of Appendicitis in Children,” Pediatric Emergency Care 23, no. 2 (2007): 69–76, 10.1097/PEC.0b013e31802d1716. 17351404

[4] B. Cohen , J. Bowling , P. Midulla , et al., “The Non‐Diagnostic Ultrasound in Appendicitis: Is a Non‐Visualized Appendix the Same as a Negative Study?,” Journal of Pediatric Surgery 50, no. 6 (2015): 923–927, 10.1016/j.jpedsurg.2015.03.012. 25841283

[5] V. G. Nasr , L. W. Markham , M. Clay , et al., “Perioperative Considerations for Pediatric Patients With Congenital Heart Disease Presenting for Noncardiac Procedures: A Scientific Statement From the American Heart Association,” Circulation. Cardiovascular Quality and Outcomes 16, no. 1 (2023): e000113, 10.1161/HCQ.0000000000000113. 36519439

[6] B. D. Thanopoulos , F. A. Hakim , A. Hiari , G. S. Tsaousis , C. Paphitis , and Z. M. Hijazi , “Patent Ductus Arteriosus Equipment and Technique. Amplatzer Duct Occluder: Intermediate‐Term Follow‐Up and Technical Considerations,” Journal of Interventional Cardiology 14, no. 2 (2001): 247–254, 10.1111/j.1540-8183.2001.tb00743.x. 12053313

[7] C. Sohrabi , G. Mathew , N. Maria , et al., “The SCARE 2023 Guideline: Updating Consensus Surgical CAse REport (SCARE) Guidelines,” International Journal of Surgery 109, no. 5 (2023): 1136–1140, 10.1097/JS9.0000000000000373. PMC10389401 37013953

[8] M. K. Mittal , P. S. Dayan , C. G. Macias , et al., “Performance of Ultrasound in the Diagnosis of Appendicitis in Children in a Multicenter Cohort,” Academic Emergency Medicine 20, no. 7 (2013): 697–702, 10.1111/acem.12161. PMC5562364 23859583

[9] S. A. Nah , S. S. Ong , W. X. Lim , S. K. Amuddhu , P. H. Tang , and Y. Low , “Clinical Relevance of the Nonvisualized Appendix on Ultrasonography of the Abdomen in Children,” Journal of Pediatrics 182 (2017): 164–169.e1, 10.1016/j.jpeds.2016.11.062. 28010937

[10] Expert Panel on Pediatric Imaging , G. C. Koberlein , A. T. Trout , C. K. Rigsby , et al., “ACR Appropriateness Criteria Suspected Appendicitis‐Child,” Journal of the American College of Radiology 16, no. 5S (2019): S252–S263, 10.1016/j.jacr.2019.02.022. 31054752

[11] E. H. Dibble , D. W. Swenson , C. Cartagena , G. L. Baird , and T. W. Herliczek , “Effectiveness of a Staged US and Unenhanced MR Imaging Algorithm in the Diagnosis of Pediatric Appendicitis,” Radiology 286, no. 3 (2018): 1022–1029, 10.1148/radiol.2017162755. 29156146

[12] S. Di Saverio , M. Podda , B. De Simone , et al., “Diagnosis and Treatment of Acute Appendicitis: 2020 Update of the WSES Jerusalem Guidelines,” World Journal of Emergency Surgery 15, no. 1 (2020): 27, 10.1186/s13017-020-00306-3. PMC7386163 32295644

[13] Z. I. Willis , E. M. Duggan , B. T. Bucher , et al., “Effect of a Clinical Practice Guideline for Pediatric Complicated Appendicitis,” JAMA Surgery 151, no. 5 (2016): e160194, 10.1001/jamasurg.2016.0194. PMC6478156 27027263

[14] J. R. Robinson , E. B. C. Avritscher , J. C. Gay , et al., “Measuring the Value of a Clinical Practice Guideline for Children With Perforated Appendicitis,” Annals of Surgery 266, no. 1 (2017): 195–200, 10.1097/SLA.0000000000001946. PMC10210014 27501175

[15] W. Wilson , K. A. Taubert , M. Gewitz , et al., “Prevention of Infective Endocarditis: Guidelines From the American Heart Association: A Guideline From the American Heart Association Rheumatic Fever, Endocarditis, and Kawasaki Disease Committee, Council on Cardiovascular Disease in the Young, and the Council on Clinical Cardiology, Council on Cardiovascular Surgery and Anesthesia, and the Quality of Care and Outcomes Research Interdisciplinary Working Group,” Circulation 116, no. 15 (2007): 1736–1754, 10.1161/CIRCULATIONAHA.106.183095. 17446442

[16] W. R. Wilson , M. Gewitz , P. B. Lockhart , et al., “Prevention of Viridans Group Streptococcal Infective Endocarditis: A Scientific Statement From the American Heart Association,” Circulation 143, no. 20 (2021): e963–e978, 10.1161/CIR.0000000000000969. 33853363

[17] V. Delgado , N. Ajmone Marsan , S. de Waha , et al., “2023 ESC Guidelines for the Management of Endocarditis,” European Heart Journal 44, no. 39 (2023): 3948–4042, 10.1093/eurheartj/ehad193. 37622656

[18] M. M. Yilmazer and T. Meşe , “Infective Endocarditis Developing Early After Percutaneous Closure of a Patent Ductus Arteriosus in a Child Using the Amplatzer Duct Occluder II,” Cardiology in the Young 30, no. 4 (2020): 591–593, 10.1017/S1047951120000542. 32188522

